# The Use of Engineered Plant Viruses in a *Trans-*Kingdom Silencing Strategy Against Their Insect Vectors

**DOI:** 10.3389/fpls.2020.00917

**Published:** 2020-07-08

**Authors:** Anna Kolliopoulou, Dimitrios Kontogiannatos, Luc Swevers

**Affiliations:** ^1^Institute of Biosciences & Applications, National Centre for Scientific Research “Demokritos”, Agia Paraskevi, Greece; ^2^Department of Biomedical Sciences, University of West Attica, Egaleo, Greece

**Keywords:** VIGS, VDPS, plant virus, insect virus, insect vector, *trans-*kingdom

## Abstract

Plants, plant viruses, and their vectors are co-evolving actors that co-exist and interact in nature. Insects are the most important vectors of plant viruses, serving as both carriers and hosts for the virus. This *trans-*kingdom interaction can be harnessed for the production of recombinant plant viruses designed to target insect genes via the RNAi machinery. The selection of the adequate viruses is important since they must infect and preferentially replicate in both the host plant and the insect vector. The routes of transmission that determine the extent of the infection inside the insect vary among different plant viruses. In the context of the proposed strategy, plant viruses that are capable of transversing the insect gut-hemocoel barrier and replicating in insect tissues are attractive candidates. Thus, the transmission of such viruses in a persistent and propagative manner is considered as a prerequisite for this strategy to be feasible, a characteristic that is found in viruses from the families *Bunyaviridae*, *Reoviridae*, and *Rhabdoviridae*. In addition, several RNA viruses are known that replicate in both plant and insect tissues via a yet unclarified transmission route. In this review, advances in knowledge of *trans-*kingdom transmission of plant viruses and future perspectives for their engineering as silencing vectors are thoroughly discussed.

## Introduction

Studies on plant viruses’ biology have shown their dependence on a plethora of vector organisms for their transmission to a new host. This vector repertoire involves insects, mites, nematodes, plasmodiophorids, and fungi (Bragard et al., 2013; Blanc et al., 2014). Although differences exist between vectors, the plant virus transmission cycle includes certain standard steps that seem to apply to almost all occasions. In the case of an insect vector, for example, (1) an infected plant is first detected as a source of food, (2) the insect feeds from the plant, (3) it acquires the virus, (4) as a carrier of the virus it can transport it, and (5) in the search for a new food source, the virus is transmitted to the next plant that the insect vector selects to feed from (Whitfield and Rotenberg, 2016).

However, different strategies (Table 1) are employed during plant virus transmission by insect vectors that are distinguished by the acquisition time and retention period of the virus by the vector (Nault, 1997). Non-persistently or semi-persistently transmitted viruses have a half-life of minutes to hours and typically involve temporary attachment to the stylet or the foregut in hemipteran insects (Blanc et al., 2014). By contrast, viruses that are persistently transmitted cross the midgut barrier and accumulate in the salivary gland, while their half-life of retention by the vectors can last from days to months (Nault, 1997; Blanc et al., 2014). Among persistently infecting viruses, plant viruses exist that also replicate in their insect vectors, a strategy called “propagative.” While the distribution of propagative viruses among insect vectors may be restricted (Hogenhout et al., 2008; Bragard et al., 2013), they raise particular interest from the biotechnological viewpoint. More specifically, genomes of propagative viruses have the potential to be engineered into agents that cause gene silencing in the insect vectors by the RNAi mechanism, which could be developed into an environmentally safe strategy to control the vectors.

**TABLE 1:** Examples of plant viruses and their insect vectors with different transmission strategies.

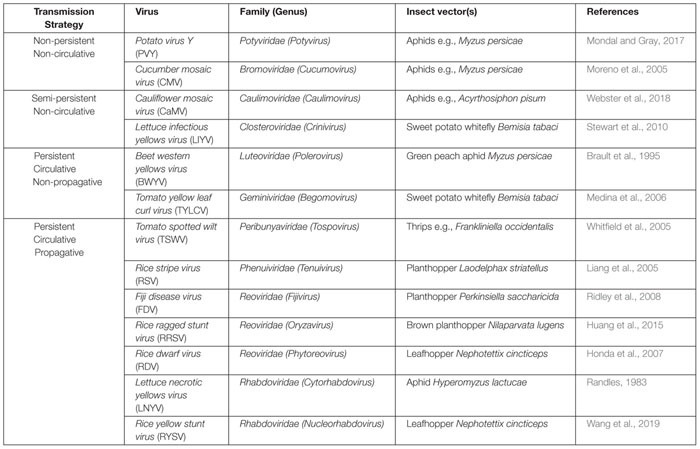

**FIGURE 1:**
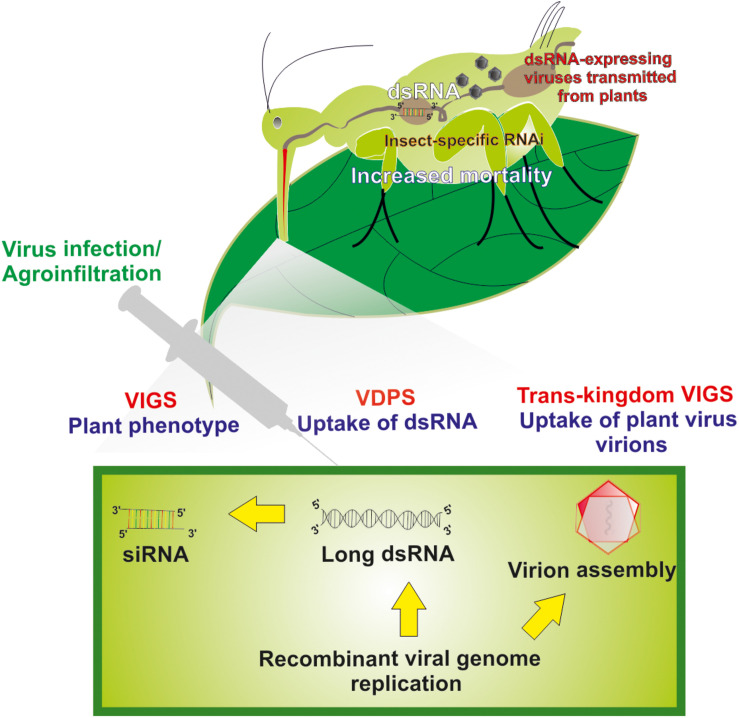
Long dsRNA production and virus-mediated gene silencing in plant cells and insect cells. Transgenic viruses are being transmitted in plants via agroinfiltration and/or injection. After infection, replication of the viral genome’s results in the production of long dsRNAs. Virus-based dsRNAs are being processed to siRNAs by the plant’s core RNAi machinery causing gene-specific phenotypes in plants (virus-induced gene silencing, VIGS). Unprocessed insecticidal long dsRNAs produced in plant cells that are being taken up by insects also can cause insect-specific RNAi that leads to increased mortality rates (virus-dependent dsRNA production, VDPS). For particular instances, assembled virions in plant cells can also be taken up by insects and, during replication in insects, release insecticidal dsRNAs that cause insect-specific RNAi and increased mortality rates (*Trans-*kingdom VIGS, TK-VIGS).

When viruses are used as viral dsRNA-producing systems (VDPS; see also further below), two main strategies can be followed regarding the introduction of the “silencing” fragment into the viral genome, as described for plant closteroviruses (Qiao and Falk, 2018): (1) An “add-a-gene” strategy that, in simple words, involves the insertion of a foreign sequence in a particular position so that it minimally interferes with the existing viral sequences. As it has been suggested, insertion near the 3′ terminus of an ORF may enhance the production of the new fragment during transcription (Navas-Castillo et al., 1997). (2) A “gene replacement” strategy of a viral genomic part encoding a non-essential protein. This second strategy is often used; however, it has been criticized for reduced efficiency compared to the previous one (Kiss et al., 2013). Because of ease of manipulation, VDPS employing plant viral vectors can prove useful as a fast-track approach to identify suitable target genes to cause toxic effects in target insects following silencing by RNAi (Kolliopoulou et al., 2017). Furthermore, similar methods of engineering of viral genomes can be used to create recombinant plant viruses that can trigger RNAi effects in insect vectors by a strategy named as “*Trans-*kingdom virus-induced gene silencing” (TK-VIGS) (Figure 1), as outlined further below.

## Routes of Transmission

Plant viruses that are known to be transmitted via particular insect families have been observed to follow diverse routes in their vectors’ bodies (Hogenhout et al., 2008; Blanc et al., 2014). This diversification in the mode of transmission can be attributed to differences in the virus genomic and structural properties, as well as to physiological and anatomical variations among insect vectors belonging to distinct genera and species (Bragard et al., 2013). A general classification of these modes of transmission has led to the establishment of three main categories that differentiate one from another regarding the time window that they can be transmissible by the insect vector to a new plant. This classification includes the non-persistent, the semi-persistent and the persistent types (Fereres and Raccah, 2015; Dietzgen et al., 2016), as discussed below. Examples of the different transmission strategies are displayed in Table 1.

In all the cases, for vectors with piercing-probing mouthparts (Hemiptera and Thysanoptera), the virus is acquired during the probing and feeding activity. In the case of the non-persistent way of transmission, plant virus acquisition by stylet piercing of plant tissue is followed by transmission to the next plant by piercing within a period of seconds to minutes. This non-persistent strategy is mainly used when aphids repetitively puncture cells during the probing of plant tissue en route to feeding with phloem sap (Stafford et al., 2012; Nalam et al., 2019). For plant virus transmission in a semi-persistent manner, the virus is known to be retained in the more proximal part of the feeding apparatus, the acrostyle, or to become bound to the chitin lining of the foregut (Killiny et al., 2016; Webster et al., 2018). The involvement of specific viral capsid proteins aiding the transmission process has been reported for the whitefly vector *Bemisia tabaci* and the *Lettuce infectious yellow virus* (LIYV) (Stewart et al., 2010; Chen et al., 2011). In that case, the virus can be transmitted over a period from several hours to days to the next target plant. Both cases of non-persistent and semi-persistent transmission are also described as non-circulative, since upon entry the plant virus is localized only at specific spots on the vector’s body (i.e., stylet, gut chitin lining) and does not cross the insect gut barrier in order to circulate to other tissues.

However, the most interesting category for biotechnological applications is that of plant viruses that are transmitted in a persistent manner, with considerable variation in terms of transmission window. This transmission route is also characterized as circulative, because the acquired virus must transverse the gut epithelium from the alimentary canal to other tissues and organs, so that it can finally reach the salivary glands. Depending on the particular plant virus–insect interaction, the transmission process may rely on specific viral proteins. Extended duration of feeding on infected plant phloem (hours), as well as prolonged retention time and a latent period, is required in order to obtain adequate transmission efficiency of the virus. Circulative plant viruses that do not replicate in the insect vectors include luteovirids, geminiviruses, and nanoviruses and are transmitted by aphids, whiteflies, and leafhoppers (Nault, 1997; Hogenhout et al., 2008). The capacity of luteoviruses to transverse the midgut epithelium has raised interest for biotechnological applications such as a new strategy to deliver peptide toxins into the hemocoel of aphids after feeding (Bonning et al., 2014).

On the other hand, viruses exist that are able to replicate and systemically invade insect tissues before reaching the salivary gland (thus taking up a propagative strategy), such as those belonging to *Reoviridae*, *Bunyaviridae*, and *Rhabdoviridae* families, as well as the *Bunyaviridae*-related genus *Tenuivirus* (Whitfield et al., 2015; Dietzgen et al., 2016). In addition to their circulation between plant and insect hosts, many propagative viruses can also be transmitted transovarially (vertically) to the insect’s offspring (Nault, 1997; Hogenhout et al., 2008). Plant viruses with a propagative transmission strategy in their insect vectors are discussed in more detail below.

## Which Insect Vectors?

By definition, plants lack any ability to move and they depend on their root system that provides connection with the soil in order for a plant to feed, grow, and remain alive. So, for any virus that inhabits a plant, it is normal to rely on mobile vectors so that it is transmitted to other plant hosts.

Among the 32 orders of Insecta, seven of them have been found to encompass vectors for plant virus transmission. Most of these vector insects belong to Hemiptera (300) and Thysanoptera (6), which are characterized by pierce-sucking mouthparts (Fereres and Raccah, 2015). It must be stressed that Hemiptera are by far the most important vector group, since they cause minimal damage while feeding and leaving cells alive for virus to replicate while probing. On the contrary, Thysanoptera tend to provoke extensive cell damage during ingestion probes, thus hampering viral replication (Stafford et al., 2012). The anatomy of the mouthparts of these insects plays an important role in the virus transmission from plant to plant, as piercing of the epidermis that is covered by an impermeable cuticle allows for the virus to be delivered intracellularly and maintains the integrity of the plant cell (Fereres and Raccah, 2015). Thrips is a characteristic type of thysanopteran insects, with 14 species being vectors of the plant-infecting *Tospovirus* genus (*Bunyaviridae*) (Riley et al., 2011; Badillo-Vargas et al., 2015).

More vectors have been observed in other orders too (i.e., Coleoptera, Orthoptera, Lepidoptera, Diptera, and Dermaptera) (Fereres and Raccah, 2015), in which however different ways of transmission are utilized that lie beyond the scope of this mini review.

## Which Viruses?

An important feature that defines the suitability of plant viral vectors for the delivery of the dsRNA silencing signal to their target insect regards the ability of the virus to replicate or not in the insect cells. Most plant viruses are not able to replicate in their insect vectors, so they can only be considered as vehicles for transfer of dsRNA that has already been synthesized inside the plant to the insect. On the other hand, replicating viruses not only are able to transverse the gut-hemocoel barrier (as “persistent circulative” viruses mentioned earlier) but also adopt a propagative strategy, which means that dsRNA intermediates are produced during their life cycle.

The viruses adaptable for gene silencing strategy, i.e., circulative-propagative viruses that do replicate in the insect body before transmission, are members of the families of *Bunyaviridae* [segmented ambisense ssRNA genome, such as *Tomato spotted wilt virus* (TSWV) whose main vector is the western flower thrips *Frankliniella occidentalis*], *Reoviridae* [segmented dsRNA genome, such as *Rice dwarf virus* (RDV) transmitted by the leafhopper *Nephotettix cincticeps*], and *Rhabdoviridae* [(−)ssRNA genome, such as *Maize mosaic virus* (MMV) vectored by the planthopper *Peregrinus maidis*]. Also *Tenuivirus* genus [(−)ssRNA genome, such as *Rice stripe virus* (RSV) and its vector planthopper *Nilaparvata lugens*] belong to this type of viruses (Whitfield et al., 2015; Dietzgen et al., 2016). Interestingly, as it will be explained later, *Tymoviridae* and *Nodaviridae* virus families that are known to have multiple kingdom hosts have already been proposed too as possible gene silencing vehicles via employment of reverse genetics techniques by taking also advantage of the existing interplay between plants and insects (Katsuma et al., 2005; Taning et al., 2018). Rhabdoviruses and bunyaviruses are the only enveloped plant viruses while reoviruses, tenuiviruses, tymoviruses, and nodaviruses do not contain a lipid envelope such as the other plant viruses that do not have a propagative transmission strategy (Hogenhout et al., 2008).

On the other hand, viruses that circulate in the insect body but in a non-propagative, thus not-replicative, manner includes members of the families of *Geminiviridae* [single or bipartite (+)ssDNA genome, such as *Tomato yellow leaf curl virus* (TYLCV) transmitted by different whitefly species], *Luteoviridae* [(+)ssRNA genome, such as *Pea enation mosaic virus* (PEMV) transmitted by the aphid *Acyrthosiphon pisum*], and *Nanoviridae* [multipartite (+)ssDNA genome, such as *Banana bunchy top virus* (BBTV) vectored by the aphid *Pentalonia nigronervosa*].

It needs to be stressed that a considerable amount of propagative plant viruses are strictly associated with particular insect vector families (Nault, 1997; Hogenhout et al., 2008). All tospoviruses are transmitted by thrips vectors; reoviruses of the *Phytoreovirus* genus and tymoviruses are transmitted by leafhoppers (Cicadellidae); and reoviruses of the genera *Fijivirus* and *Oryzavirus* as well as tenuiviruses are transmitted by planthoppers (Delphacidae). A notable exception, however, is the rhabdoviruses that can be transmitted either by aphids, leafhoppers and planthoppers (Nault, 1997; Hogenhout et al., 2008).

## Production of vsiRNAs as a Sign of RNAi Mechanism Activation

Double-stranded RNA molecules are expected to be produced by propagative viruses, during their replication or transcription steps inside the insect host’s body after infection. Reoviruses possess a dsRNA multisegmented genome from which mRNA can be transcribed. For the production of new dsRNA molecules, (+)RNA strands are encapsidated in a subviral particle, where they are transcribed into (−)RNAs, so that dsRNA is then produced by base-pairing of the complementary (+) and (−) strands. In the case of single-stranded or ambisense RNA viruses, intermediate replicative steps normally may also lead to the temporary production of dsRNA molecules. Engineering of plant viruses that possess single-stranded RNA genomes with appropriately designed sequences in order to transcribe RNA molecules containing self-hybridizing regions would lead to the formation RNA hairpin structures. These RNA hairpins could then be processed in dsRNAs and siRNAs by the RNAi machinery of the insect vector. Also, regarding DNA viruses inverted repeats can be introduced in their sequence so that RNA hairpins with dsRNA structures can be formed upon transcription.

As the replication of propagative plant viruses is expected to lead to the production of viral short-interfering RNAs (vsiRNAs) in the insect vector, it can be assumed that the RNAi silencing machinery is turned on upon such an infection. Several examples of vsiRNA production in insects during plant virus infection have been reported in the literature, such as the infection of the small brown planthopper *Laodelphax striatellus* by the *Rice black-streaked dwarf virus* (RBSDV, *Reoviridae*) or RSV (*Tenuivirus*) (Xu et al., 2012; Li et al., 2013; Yang et al., 2018), of the white-backed planthopper *Sogatella furcifera* by RBSDV (Wang et al., 2016), in the zigzag leafhopper *Recilia dorsalis* by the *Rice gall dwarf virus* (RGDV, *Reoviridae*) (Lan et al., 2016a), and in the planthopper *Delphacodes kuscheli* by *Mal de Río Cuarto virus* (MRCV, *Reoviridae*) (De Haro et al., 2017). Furthermore, vsiRNA production was observed in the glassy-winged sharpshooter *Homalodisca vitripennis* (Cicadellidae) after infection with *H. vitripennis reovirus* (HoVRV) (*Phytoreovirus* genus) (Nandety et al., 2013) although a plant host for this virus was not identified (Stenger et al., 2009).

However, while deep sequencing efforts have detected vsiRNAs of propagative plant viruses in hemipteran vectors, their functionality (capacity of gene silencing) remains largely unexplored. Thus, a systematic effort needs to be initiated regarding the role of RNAi as an antiviral defense mechanism against replicating plant viruses in insect vectors, which will include the effect of knockdown of the RNAi machinery on viral replication [of which limited data are available (Lan et al., 2016a, b)] and the possible existence of Viral Suppressors of RNAi (VSR) genes in viral genomes [which are known to act within plants (Cao et al., 2005; Xiong et al., 2009)]. In plants, on the other hand, RNAi has been used successfully to control plant viruses that are transmitted by insect vectors in a circulative propagative manner [e.g., RBSDV (Shimizu et al., 2011)].

## Examples of Engineered Plant Viruses to Target Their Insect Vectors: From VDPS to *Trans-*Kingdom VIGS

As a first straightforward application of engineered plant viruses causing gene silencing in insect vectors, the VDPS system (Kumar et al., 2012) was developed and its silencing efficiency was shown to compare favorably with conventional plant-mediated RNAi (PMRi; using transgenes to express dsRNA molecules). As will be explained in this section, the paradigm of VDPS was later expanded and further elaborated by the use of more sophisticated engineering concepts in several virus–vector combinations.

In the case of Hemiptera, an interesting example of efficient application of recombinant VIGS (here used as VDPS) technology to combat insect vectors was the construction of recombinant *Tobacco mosaic virus* (TMV, *Virgaviridae*) that produced RNAs in sense or antisense orientation targeting *actin*, *chitin synthase 1*, and *V-ATPase* genes of the hemipteran pest *Planococcus citri* that were initially inoculated to *Nicotiana benthamiana* plants through agroinfiltration. This method was successful in causing silencing of the above-mentioned genes of the insect vector (Khan et al., 2013) that is known to facilitate the transmission of *Grapevine leafroll associated virus 3* (GLRaV-3, *Closteroviridae*) among grapevine plants (Cabaleiro and Segura, 1997). Another hemipteran, the phloem-feeding *Bactricera cockerelli* was tested as a possible target by recombinant TMV viruses in which sequences against *BC*-*actin* and *BC*-*V-ATPase* genes were cloned, primarily infecting tomato (*Solanum lycopersicum*), tomatillo (*Physalis philadelphica*), and tobacco (*Nicotiana tabacum*) plants. In the same study, two other viruses (*Potato virus X*, PVX, *Alphaflexiviridae*; *Tobacco rattle virus*, TRV, *Virgaviridae*) were tested (Wuriyanghan and Falk, 2013). A few years later, however, PVX was repeatedly reported as an efficient and easy tool for *in planta* RNAi induction against the hemipteran phloem-feeding pest mealybug *Phenacoccus solenopsis* (by agroinfiltrating *N. tabacum* plants). In these studies, the *Bursicon*, *V-ATPase* and *Chitin synthase 1* genes of the insect vector were targeted, as proven by decrease in expression and increase in population mortality as well as other phenotypic effects (Khan et al., 2015, 2018). A virus of the same genus, *Alternanthera mosaic virus* (AltMV, *Alphaflexiviridae*), was used to create an RNAi vector for the silverleaf whitefly *Bemisia tabaci* where numerous cDNA clones could be then inserted and used as a screening method for finding the ideal target gene (Ko et al., 2015). Also *Citrus tristeza virus* (CTV, *Closteroviridae*) was effectively used to silence the *altered wing disc* (*Awd*) gene in CTV-based RNAi assays against the hemipteran phloem-sap sucking insect *Diaphorina citri*, although this particular virus is known to normally encode for three different silencing suppressors. Interestingly, these experiments involved a few additional steps, as CTV constructs were first agroinfiltrated into *N. benthamiana* plants, and virions produced there were then isolated and inoculated to *Citrus macrophylla* plants to trigger silencing in its target insect *D. citri* (Hajeri et al., 2014).

In the above examples, plant viruses were engineered as a VDPS to produce dsRNA in plant tissues at high levels to cause gene silencing and mortality in insects after feeding. The RNAi effects in the insect vectors were not caused by plant virus replication within the vectors but by the large amounts of dsRNA produced in the plant cells. VDPS is not considered a static system since it can be envisioned that the VDPS systems and the viruses that sustain them are spread through the plant populations by taking advantage of the virus transmissibility by insect vectors (Qiao and Falk, 2018). However, VDPS is not necessarily dependent on hemipteran vectors; for example, TRV is transmitted by nematode vectors (Ploeg et al., 1993) while PVX is only mechanically transmitted (Franc and Banttari, 2001). In such cases, spread in the field can be more limited and will depend on the transmission strategy of the virus that is used in the VDPS. By its nature, VDPS is also not limited to affect hemipteran insect vectors but can be employed to control agricultural pests belonging to different insect orders such as Lepidoptera (Kumar et al., 2012; Bao et al., 2016).

A more challenging category of viruses are those that infect and replicate in hosts belonging to different kingdoms, such as viruses of the *Tymoviridae* family that are known to infect plants but are also non-persistently transmitted by phloem-sucking insects (Martelli et al., 2002a, b). However, “tymovirus-like viruses” have the ability to replicate in insect tissues. *Culex Tymoviridae-like virus* (CuTLV) was isolated a few years ago from mosquitoes in China and found to present high sequence similarities with other plant-infecting *Tymoviridae* genera (Wang et al., 2012). Moreover, recently a new *Tymoviridae-like virus* was identified in *Culex quinquefasciatus* mosquitoes in Mexico (Charles et al., 2019). However, both these viruses have not been assigned officially to the *Tymoviridae* family, while it remains to be investigated whether they can be transmitted and possibly replicate in plants too (Wang et al., 2012; Charles et al., 2019). Similarly, sequencing has revealed the presence of *Bee macula-like virus* (BeeMLV) and *Bombyx mori macula-like virus* (BmMLV) in honeybee and silkworm samples, with the genus *Maculavirus* being one of the main genera of *Tymoviridae* family, thus meaning a possible interplay of these virus between hosts of plant and insect origin (Katsuma et al., 2005; De Miranda et al., 2015). Up to now, it remains unclear whether a particular “tymovirus-like virus” can replicate in both insect and plant tissues and whether host switching (from plant to insect and vice versa) occurs frequently in nature. Interestingly, *Flock house virus* (FHV) is an insect pathogenic virus that belongs to the *Nodaviridae* family, but can also replicate and produce functional virions in plants (Selling et al., 1990). Engineered transencapsidated FHV has been efficiently packaged upon agroinfiltration in plant cells and used as vehicle for transfer as vaccine in mammalian cells (Zhou et al., 2015). Also, the modification of FHV genome for silencing specific genes was shown to lead to targeted gene suppression and mortality in insects *in vivo* and *in vitro* (Taning et al., 2018). Therefore, FHV could be engineered to carry purposely designed sequences against an insect host, so that it was ideally produced in a plant system and transmitted to insects in order to control a dangerous insect pest population. However, also in the case of nodaviruses the mode and efficiency of transmission between plants and insects needs much more investigation before the proposed strategy can be employed.

*Sonchus yellow net rhabdovirus* (SYNV) is another plant virus for which a reverse genetics system became available (Wang et al., 2015). Because SYNV presumably is transmitted by aphid vectors in a circulative-propagative manner, the potential exists for the usage of recombinant SYNV as a gene silencing inducing agent in aphids. For instance, recombinant SYNV viruses that target essential genes of the aphid vectors could be produced in plants and deployed to infect aphid vectors and cause toxic effects following replication and dsRNA production, an approach termed *Trans-*kingdom virus-induced gene silencing (TK-VIGS; Figure 1). However, reverse engineering of SYNV remains a major challenge because of its large size and possible pathological effects caused during infection of plant tissues. While this approach is very attractive conceptually, major engineering efforts are considered necessary to make it a viable tool.

## Conclusion

The strategy of modifying plant viruses in order to produce novel tools that will offer the potential of virus-mediated gene silencing in insects is an attractive option for pest control. Because RNA molecules are considered safer than proteins (Petrick et al., 2013; Ramon et al., 2014; Casacuberta et al., 2015), the strategy of causing RNAi effects may be looked upon favorably by regulatory bodies. The engineering of plant viruses to silence genes in their insect vectors offers advantages such as the specificity of their target gene silencing, as nowadays RNAi has turned into a well-established and feasible control technique. For instance, VDPS is expected to accelerate the screening procedures for candidate genes, and thus for target sequences, in the frame of designing a well-orchestrated RNAi-mediated agricultural pest control strategy (Hajeri et al., 2014). Obviously, compared to other methods like the development of transgenic plants, VDPS is considered as an impressively rapid and cost-effective alternative technique (Bao et al., 2016). However, a relevant concern regarding the use of this technique is that recombinant plant viruses are considered to include a risk of being quite unstable, since the insertion of duplicated sequences increases the possibility of homologous recombination incidents, therefore stressing a tendency for them to return to the wild type (Donson et al., 1991; Qiao and Falk, 2018).

In this review, we propose a more radical strategy of using viral vectors to cause gene silencing in insects, which is called *Trans-*kingdom VIGS (Figure 1). The challenge will be to produce plant viruses that cause minimal damage in host plants but trigger cellular damage and mortality following infection and replication in insect vectors after transmission. Because the viruses replicate in both types of hosts, a high efficiency of transmission of RNAi triggers is expected. Furthermore, because propagative plant viruses can be transmitted vertically in insect vectors, the silencing effect may become amplified along the next generation.

In comparison to VDPS, however, *Trans-*kingdom VIGS has some restrictions. VDPS can be harnessed to control not only hemipteran vectors but also other agricultural pests, most notably lepidopteran larvae, because it is based on production of dsRNAs in plant tissues. In *Trans-*kingdom VIGS, dsRNAs are produced in insect (hemipteran) vectors during their transmission and its efficiency therefore is limited to particular insect species that are competent hosts for propagative transmission. *Trans-*kingdom VIGS is therefore more likely to be developed as a “tailor-made” application for the control of particular plant virus–insect vector combinations.

To achieve such goals, much more knowledge needs to be acquired for the establishment of reverse genetics systems for propagative plant viruses and the stimulation of the transmission between plant hosts and insect host/vectors. Furthermore, viruses (e.g., FHV) exist that have a very broad host range and that can replicate in both plants and insects also deserve renewed attention to see if they can be adapted to the strategy of *Trans-*kingdom VIGS.

## Author Contributions

AK, DK, and LS conceived the idea, designed the study, and wrote the manuscript. DK prepared the figures of the study. All authors read and approved the final version of the manuscript.

## Conflict of Interest

The authors declare that the research was conducted in the absence of any commercial or financial relationships that could be construed as a potential conflict of interest.
